# ω-3 Fatty Acids and Cardiovascular Diseases: Effects, Mechanisms and Dietary Relevance

**DOI:** 10.3390/ijms160922636

**Published:** 2015-09-18

**Authors:** Hanne K. Maehre, Ida-Johanne Jensen, Edel O. Elvevoll, Karl-Erik Eilertsen

**Affiliations:** Norwegian College of Fishery Science Faculty of Biosciences, Fisheries and Economics, UIT The Arctic University of Norway, N-9037 Tromsø, Norway; E-Mails: ida-johanne.jensen@uit.no (I.-J.J.); edel.elvevoll@uit.no (E.O.E.); karl-erik.eilertsen@uit.no (K.-E.E.)

**Keywords:** *n*-3 fatty acids, eicosapentaenoic acids (EPA), docosahexaenoic acid (DHA), lipid peroxidation, cardiovascular diseases, seafood, supplements

## Abstract

ω-3 fatty acids (*n*-3 FA) have, since the 1970s, been associated with beneficial health effects. They are, however, prone to lipid peroxidation due to their many double bonds. Lipid peroxidation is a process that may lead to increased oxidative stress, a condition associated with adverse health effects. Recently, conflicting evidence regarding the health benefits of intake of *n*-3 from seafood or *n*-3 supplements has emerged. The aim of this review was thus to examine recent literature regarding health aspects of *n*-3 FA intake from fish or *n*-3 supplements, and to discuss possible reasons for the conflicting findings. There is a broad consensus that fish and seafood are the optimal sources of *n*-3 FA and consumption of approximately 2–3 servings per week is recommended. The scientific evidence of benefits from *n*-3 supplementation has diminished over time, probably due to a general increase in seafood consumption and better pharmacological intervention and acute treatment of patients with cardiovascular diseases (CVD).

## 1. Introduction

The story of ω-3 fatty acids (*n*-3 FA) and their impact on health and diseases began in the 1970s when it was discovered that the Inuit in Greenland had a markedly lower incidence of cardiovascular diseases (CVD) than the Danish population. The Danish researchers Bang and Dyerberg conducted several studies comparing plasma lipid profiles and food habits between the two populations. Their main conclusion was that there was a correlation between diet compositions in the two populations and the frequency of CVD. A large difference in intake of the long-chain polyunsaturated fatty acids (PUFA) eicosapentaenoic acids (EPA, C20:5, *n*-3) and docosahexaenoic acid (DHA, C22:6, *n*-3) was concluded to be the main explanatory factor for this phenomenon [1,2,3,4]. Recently, several papers have been published raising questions about the conduction of Bang and Dyerberg’s research. The main issues in these papers are that the systems for health monitoring and cause of death registration in Greenland at the time were inadequate due to the dispersed settlements [5,6].

Nevertheless, numerous studies on the relationship between seafood or EPA + DHA supplements and several diseases have been performed and positive effects of a high intake have been documented for a range of different medical conditions, in particular conditions related to inflammatory processes.

However, there is a possible drawback associated with intake of refined and concentrated *n*-3 PUFA. Due to their high content of double bonds, they are especially prone to lipid peroxidation. Metabolites from lipid peroxidation have been under suspicion for negatively affecting biological processes. A high intake of *n*-3 PUFA could thus be a double-edged sword.

Seafood consumption has traditionally been a robust measure in epidemiological studies, showing a clear correlation between a high seafood intake and reduced risk of CVD. Various fish oil supplements have been tested in clinical trials, giving partly conflicting results compared to the epidemiological studies. In this review, recent literature regarding different health aspects of *n*-3 PUFA intake from different sources will be discussed and possible reasons for conflicting findings between them will be addressed.

## 2. Cardiovascular Diseases

Cardiovascular disease is a collective term comprising a group of disorders of the heart and blood vessels. These diseases are the largest cause of morbidity and premature death worldwide and accounted for 17.5 million deaths, or 31% of all deaths, in 2012 [7]. The two most frequent disorders are coronary heart disease (CHD) and cerebrovascular disease (stroke), making up 42% and 38% of CVD deaths, respectively. There are several risk factors associated with the development of CVD, among them dyslipidemia, hypertension, tobacco smoking, obesity and diabetes mellitus [8]. All of these risk factors are associated with oxidative stress, which is a condition that arises when there is an imbalance between pro-oxidants (such as reactive oxygen species, ROS) and antioxidants (such as glutathione) in the body. A combination of multiple factors increases the risk considerably, the rule of thumb being the greater the level of each risk factor, the greater the total risk [9].

One of the major underlying causes for CVD is atherosclerosis, which is a complex, multifactorial and progressive inflammatory condition affecting the arteries. Intima, the inner layer of arteries, consists of endothelial cells (EC) with a vast range of metabolic and regulatory functions. These include transport of metabolic substances, regulation of vascular tone, defense against inflammation, angiogenesis and regulation of hemostasis and coagulation [10]. A key substance in these regulatory processes is the vasodilator nitric oxide (NO) and reduced bioavailability of this compound, for instance by increased oxidative stress, may lead to an activation of the EC [11], which over time may evolve into a condition called endothelial dysfunction. Activation of EC leads to an inflammatory response involving the production of a cascade of chemokines (monocyte chemoattractant protein-1 (MCP-1), macrophage colony-stimulating factor (M-CSF), interleukin-8 (IL-8) *etc*.), adhesion factors (vascular cell adhesion molecule-1 (VCAM-1), intercellular adhesion molecule-1 (ICAM-1), selectins (endothelial and platelet selectins (E-selectin and P-selectin) *etc*.) and integrins (very late antigen 1 and 4 (VLA-1 and VLA-4) *etc*.) produced by both circulating blood and endothelial cells. These released substances are involved in the concurrent processes of further recruitment of monocytes to the endothelial surface, followed by their adherence and transmigration into the vessel wall intima. The influx of monocytes is often accompanied by influx of other inflammation cells, such as T-cells, dendritic cells and mast cells. Once placed in the intima, monocytes may differentiate into macrophages influenced by pro-inflammatory cytokines (IFN-γ, IL-1β, TNFα, *etc*.). Macrophages are phagocytic cells expressing scavenger receptors for uptake of modified low density lipoprotein (LDL). In these activated macrophages, programmed to protect our bodies from danger, the normal processes for cholesterol handling and transport is impaired and accumulation of cholesteryl esters will eventually lead to the formation of foam cells, also known as fatty streaks [12]. Continued inflammatory responses may further accelerate the progression of atherosclerotic lesions. Stimulation of proliferation and migration of smooth muscle cells (SMC) may build up a large plaque inside the intima. Protease secretion by macrophages degrade extracellular matrix, such as collagen, and a fibrous cap is formed around the excess lipids. Expression of collagen degrading enzymes, such as matrix metalloproteinases (MMP) can gradually weaken the fibrous cap leading to plaque rupture and release of intracellular content into the arteries, thrombus formation and this may eventually result in myocardial infarction (MI) [13].

## 3. ω-3 Fatty Acids and Their Metabolites

### 3.1. General

The common chemical structure of FA is an aliphatic hydrocarbon chain of with a carboxylic acid group at one end and a methyl group at the other end. The length of the carbon chain normally ranges between 4 and 22 carbon atoms and they are covalently bound together with only single bonds, with one carbon-carbon double bond or with up to six double bonds. The respective fatty acids are classified as saturated, monounsaturated or polyunsaturated FA, depending on the number of double bonds in the chain. Unsaturated FA are further classified by the so-called “ω” denotation, which indicates the placement of the first double bond in the chain, counted from the methyl end of the C-chain.

Fatty acids are present in a range of different structures in the body and they also have a range of different properties. Bound in triacylglycerols (TAG) they serve as energy storage, and bound in phospholipids (PL) they serve as integral parts of cellular membranes. They are also origin for a range of compounds with specific biological functions and some of these are of particular interest when it comes to health issues and development of different medical conditions/chronic diseases.

### 3.2. Eicosanoids and Specialized Pro-Resolving Mediators (SPM)

A wide range of compounds may be enzymatically synthesized from FA containing 20 or 22 C-atoms. Several of these metabolites, such as eicosanoids and so-called specialized pro-resolving mediators (SPM), have impact on acute and chronic inflammatory responses.

Eicosanoids are hormone-like substances originating from FA containing 20 C-atoms, namely dihomo-γ-linolenic acid (DGLA, C20:3, *n*-6), arachidonic acid (AA, C20:4, *n*-6) and EPA. Eicosanoids include prostaglandins (PG), prostacyclins (PI), thromboxanes (TX) and leukotrienes (LT). All subclasses of eicosanoids may be synthesized from all of the three mentioned FA; however, nomenclature, properties and potencies depend on which FA is the origin [14]. Nomenclature of the eicosanoids are related to the number of double bonds in the structure and hence, prostaglandins derived from AA are categorized as 2-series PG, while those derived from EPA are categorized as 3-series PG. Leukotrienes from the two FA are categorized as 4-series LT and 5-series LT, respectively.

The SPM comprise lipoxins, resolvins, maresins and protectins, all inflammation-resolving compounds which can be derived from AA (lipoxins), EPA (resolvins) and DHA (resolvins, maresins and protectins) [15].

**Figure 1:**
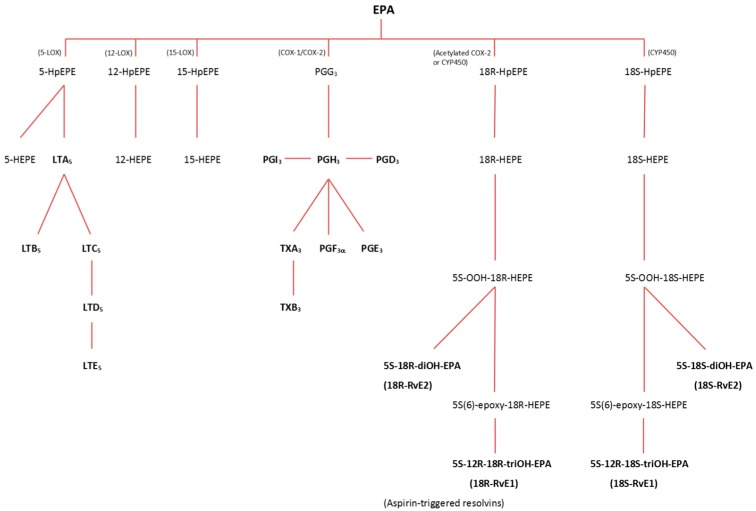
Overview of the pathways involved in the production of eicosanoids and specialized pro-resolving mediators from eicosapentaenoic acid (EPA). The figure is adapted from [16,17]. Products derived through the lipoxygenase (LOX) pathway are 5-, 12- and 15-HEPE, along with the leukotrienes A to E (LTA, LTB, LTC, LTD and LTE). Through the cyclooxygenase (COX) pathway, the prostaglandins D to I (PGD, PGE, PGF, PGG, PGH and PGI) and thromboxanes A and B (TXA and TXB) are produced. Aspirin-acetylated COX-2 catalyzes the production of aspirin-triggered resolvins E1 (18*R*-Rv1) and E2 (18*R*-Rv2), while the regular resolvins E1 (18*S*-Rv1) and E2 (18*S*-Rv2) are catalyzed by cytochrome P-450 (CYP-450). HpEPE: hydroperoxyeicosapentaenoic acids; HEPE: hydroxyeicosapentaenoic acids.

**Figure 2:**
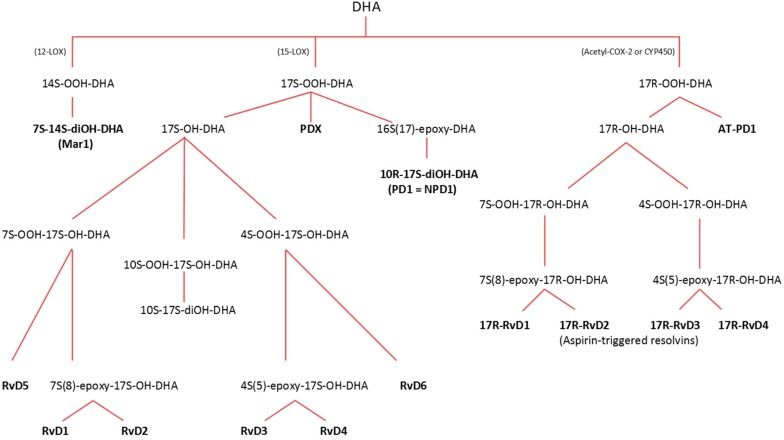
Overview of the pathways involved in the production of specialized pro-resolving mediators from docosahexaenoic acid (DHA). The figure is adapted from [17]. Through the lipoxygenase (LOX) pathway, LOX-12 catalyzes the production of maresin-1 (Mar-1), while 15-LOX catalyzes the formation of neuroprotectin D1 (NPD1), protectin DX (PDX) and resolvins D1 (17*S*-RvD1) to D6 (17*S*-RvD6). Aspirin-acetylated cyclooxygenase-2 (COX-2) and/or cytochrome P-450 (CYP-450) catalyzes the formation of aspirin-triggered resolvins D1 (17*R*-RvD1) to D4 (17*R*-RvD4), along with aspirin-triggered protectin D1 (AT-PD1).

Synthesis of all of these compounds from AA, EPA and DHA starts with the liberation of the FA from the membrane phospholipids catalysed by phospholipase A_2_. Several enzymes are involved in the following synthesis pathway (Figure 1 and Figure 2), the main classes being lipoxygenases (LOX), cyclooxygenases (COX) and cytochrome P450 (CYP) [14]. The metabolic pathways for formation of the eicosanoids and SPM are similar independent of the originating FA, and in this review, only the intermediary products of EPA and DHA will be presented.

#### 3.2.1. Lipoxygenase (LOX) Pathway

There are three major lipoxygenases (5-, 12- and 15-LOX) involved in the formation of eicosanoids and SPM [18]. These enzymes catalyse the insertion of a hydroperoxy group in the C-chain of the FA. The numbering of the LOX corresponds to the C-atom on which the insertion takes place. The resulting compounds are hydroperoxyeicosapentaenoic acids (HpEPE) which is further rapidly reduced to hydroxyeicosapentaenoic acids (HEPE).

**Figure 3:**
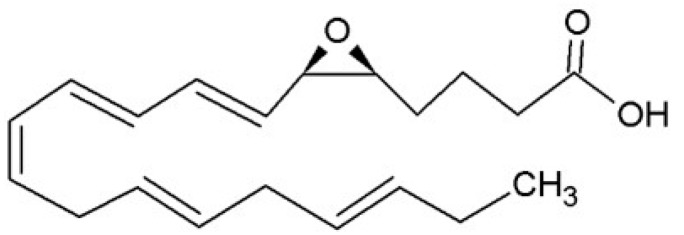
Leukotriene A_5_.

The most common products derived from EPA via the LOX-pathways are 5-series LTs. Their formation is catalysed by 5-lipoxygenase (5-LOX), which is activated by 5-lipoxygenase activating protein (FLAP) and converts EPA to LTA_5_ (Figure 3) via dehydration of the unstable 5-hydroperoxy-eicosapentaenoic acid (5-HpEPE). Several enzymatic reactions in different cells thereafter convert LTA_5_ to other LT, namely LTB_5_, LTC_5_, LTD_5_ and LTE_5_ [19].

Also, DHA may act as a substrate for different LOX pathways, forming resolvins of the d-series, along with maresin 1 and protectin D1. Maresin 1 is formed via the 12-LOX pathway, while d-series resolvins (D1–D6) and protectin D1 are formed via the LOX-15 pathway [15,17].

#### 3.2.2. Cyclooxygenase (COX) Pathway

Prostaglandins, prostacyclins and thromboxanes are formed by enzymatic conversion of EPA by prostaglandin-endoperoxidase synthase (PTGS), more commonly known as cyclooxygenase (COX). This enzyme has two main isoforms involved in the synthesis of prostaglandins, COX-1 and COX-2, having both distinct and common properties [20,21]. Also, a third COX-isoform (COX-3) has been discovered, but this isoform seems to have low or limited impact on the eicosanoid synthesis [22].

**Figure 4:**
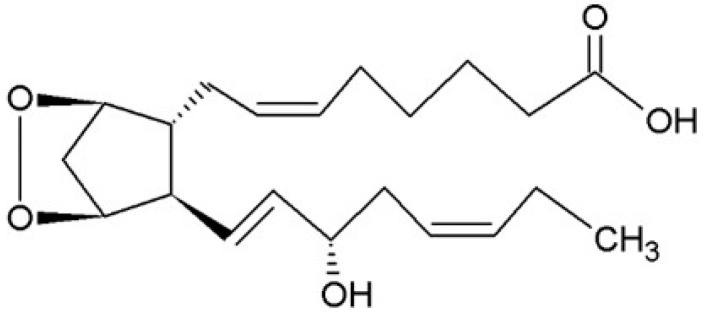
Prostaglandin G_3_.

The eicosanoid synthesis starts with the insertion of two oxygen molecules to the EPA forming a cyclopentane ring structure between C8 and C12 and an oxygen bridge between C9 and C11. In addition, a hydroperoxy group is inserted in the *S*-chirality at C15. This intermediate compound is called prostaglandin PGG_3_ (Figure 4) and is rapidly reduced to PGH_3_ by peroxidase. Tissue-specific enzymes thereafter convert PGH_3_ to the other PG, PI and TX.

Both isoforms of the COX-enzyme may be acetylated by aspirin, leaving COX-1 deactivated. Aspirin-acetylated COX-2, on the other hand, may catalyse the insertion of a hydroperoxy group in the *R*-chirality at C18 in EPA resulting in the formation of 18*R*-hydroperoxy-eicosapentaenoic acid (18*R*-HpEPE). This peroxide is then converted into aspirin-triggered resolvins E1 and E2 by several other enzymatic reactions involving peroxidases, 5-LOX and epoxidases [17]. Aspirin-acetylated COX-2 may also trigger a similar process in DHA, resulting in the formation of aspirin-triggered resolvins D1-D4 or protectin PD1 [18].

#### 3.2.3. Cytochrome P-450 (CYP) Pathway

The third enzymatic pathway important in the metabolization of LC-PUFAs is the cytochrome P-450 pathway. Here, CYP monooxidases inserts oxygen into the AA, EPA or DHA to yield either hydroxylated or epoxidized FA. Hydroxylation takes place at C19 or C20 in EPA and C21 or C22 in DHA, while epoxidation may take place at each of the double bonds in the FA [23,24].

This pathway may also promote the formation of non-aspirin-triggered resolvins from EPA, via insertion of a hydroperoxy group in the *S* chirality of C18, forming 18*S*-HpEPE. In a process similar to the one described for aspirin-triggered resolvins, peroxidases, 5-LOX and epoxidases further convert this compound into resolvins [17].

#### 3.2.4. Eicosanoids, SPM and Cellular Effects on Atherosclerosis

Eicosanoids are involved in a vast range of biological processes and the subspecies most relevant for the development of CVD are PGI, TXA and LTB. These compounds exert their effects on EC, monocytes, blood platelets and vascular smooth muscle cells (VSMC) [25], all of which are involved in the atherosclerotic process. Thromboxane A_2_ affects aggregation of platelets and is a potent vasoconstrictor, while LTB_4_ induces inflammation, leukocyte chemotaxis and adherence. Although their EPA-derived counterparts affect many of the same mechanisms, their potency is much lower and the adverse effects are not as pronounced. The third subspecies, PGI, have opposite effects as they serve as active vasodilators and inhibit platelet aggregation [26].

Only very few studies have been published showing the direct effects or mechanisms of the different eicosanoids on inflammation cascade factors and processes [27,28,29]. In contrast, as reviewed by Yates *et al.* [30], multiple cell-based studies have shown indirect effects where addition of *n*-3 FA reduces the inflammatory responses effectively by downregulating or inhibiting expression of these cascade compounds. In some comparative studies, DHA has been shown to be more effective than EPA in attenuating the effects of these compounds [31,32].

The anti-inflammatory mechanisms of the SPM vary between the different compounds, but some common features are reduction of migration of activated immune cells to the injured endothelium and stimulation of phagocytic ability of macrophages. Comprehensive overviews of the anti-inflammatory and inflammation resolving properties of the SPM are presented in several reviews [19,33].

### 3.3. Lipid Peroxidation and Its Products

**Figure 5:**
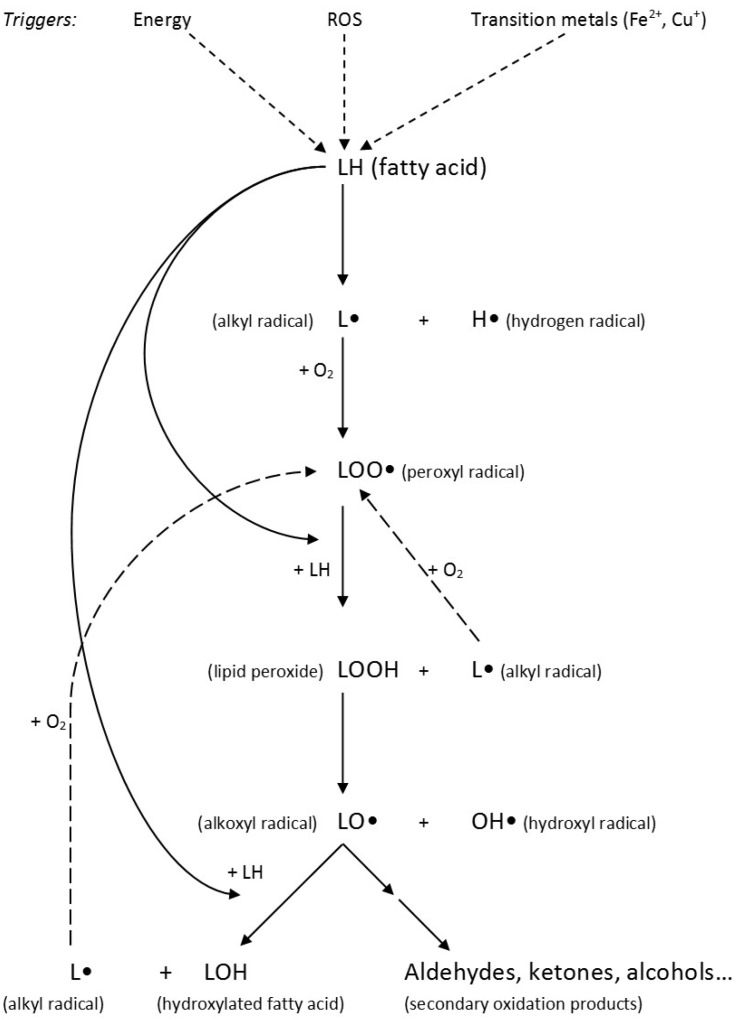
Illustration of the lipid peroxidation process. Triggered by energy, reactive oxygen species (ROS) and/or transition metals, an unstable hydrogen atom is abstracted from a fatty acid (LH), forming an alkyl radical (L•) and a hydrogen radical (H•). The alkyl radical reacts with oxygen, forming a peroxyl radical (LOO•) that further reacts with another fatty acid, forming a lipid peroxide (LOOH) and a new alkyl radical. The lipid peroxide is easily degraded into an alkoxyl radical (LO•) and a hydroxyl radical (OH•). The alkoxyl radical may either react with a new fatty acid, forming a hydroxylated fatty acid (LOH), or decompose into smaller, volatile compounds, such as aldehydes, ketones or alcohols.

Polyunsaturated fatty acids are, due to unstable double bonds, prone to oxidation. The incidence of oxidation increases with the number of double bonds present and hence, the long chain *n*-3 FA are especially exposed. Lipid oxidation may occur either in foods or endogenously and is a three-phased chain reaction divided into initiation, propagation and termination (Figure 5). The initiation phase, triggered by energy (as for instance oxygen, light energy and radiation), ROS and transition metals, starts with the abstraction of the unstable H-atom between the double bonds of the PUFA resulting in the formation of an alkyl radical (L•) and a hydrogen radical (H•). Delocalisation of the remaining double bonds result in the formation of conjugated double bonds, either in *cis* or *trans* configuration. Due to increased stability, the *trans* configuration dominates. In the propagation phase, oxygen is added to the alkyl radical, forming a peroxyl radical (LOO•). The peroxyl radical reacts with another unstable H-atom in a PUFA, forming a peroxide (LOOH) and a new alkyl radical and the chain reaction has commenced. Peroxides are known as primary oxidation products. They are unstable and easily degraded to alkoxyl radicals (LO•) and hydroxyl radicals (•OH). The alkoxyl radical can either affect the initiation phase, forming a hydroxylated FA (LOH) or it can be decomposed into smaller molecules, such as alcohols, aldehydes and ketones. The amount of radicals, peroxides and oxidation products increase exponentially up until the radicals start reacting with each other, forming stable polymeric products. This phase is known as the termination phase. All unsaturated FA may undergo this process. The products formed depend on the original FA and each of them can be the source of multiple products. The possible range of lipid peroxidation products is therefore enormous. Some of the most studied lipid peroxidation products are reactive aldehydes, such as malondialdehyde (MDA), and 4-hydroxy-2-alkenals [34,35,36]. While MDA can be derived from all FA with three or more double bonds, 4-hydroxy-2-nonenal (HNE) is derived from *n*-6 PUFA and 4-hydroxy-2-hexenal (HHE) is derived from *n*-3 PUFA.

#### Lipid Peroxidation Products and Cellular Effects on Atherosclerosis

The free radicals formed during lipid oxidation may contribute to oxidative stress, a condition which stimulates the activation of nuclear factor κB (NFκB) and subsequently the production of inflammatory chemokines, adhesion factors and cytokines [37]. In addition, both the free radicals and the primary lipid oxidation products (lipid peroxides) may accelerate further lipid peroxidation [38]. The presence of primary oxidation products in foods is not easily detected, as they are tasteless and odourless. The secondary oxidation products, however, are formed rapidly and are more easily detected in oxidized foods because of their volatility, which causes a rancid taste and odour.

In toxicology studies, HNE and HHE showed equal cytotoxic properties on rat cortical neurons and also on the depletion of the endogenous antioxidant glutathione [39]. Activation of NFκB, which stimulates the production of the previously described chemokines, adhesion factors and cytokines, has been shown for HHE [40]. In contrast, in low concentrations, 4-HHE has been shown to induce the antioxidant enzyme heme oxygenase-1 (HO-1) through activation of the nuclear factor-erythroid-2-related factor-2 (Nrf2) and thereby protect vascular cells from cytotoxicity induced by oxidative stress [41].

A property of reactive aldehydes especially relevant regarding CVD are their capability of forming covalent bonds to DNA, proteins and phospholipids [36]. After binding to the protein constituent of LDL (apoB), they contribute to oxidative modification of this particle. Oxidized LDL are triggers of endothelial dysfunction, which is crucial for the onset of atherosclerosis. They are also easily digested by macrophages and hence contribute to the development of foam cells [42] and later to fatty streaks and atherosclerotic lesions. Protein-bound HHE has also been detected in atherosclerotic lesions [43].

### 3.4. Clinical Effects of n-3 Intake on Atherosclerosis

An increased intake of *n*-3 FA has been shown to increase the phospholipid content of these FAs in both plasma membranes and red blood cells at the expense of AA. The relative proportion of EPA + DHA in the red blood cell membranes (as percentage of total FA), is termed the ω-3-index. This value has been shown to be dose-dependent on the *n*-3 FA intake [44,45] and a low index was first recognized as an independent risk marker and later as an actual risk factor for CHD death [46,47,48]. As EPA competes with AA in the COX and LOX-mediated eicosanoid production, a consequence of this is that the eicosanoid production also is shifted towards a higher level of 3-series prostaglandins and 5-series of leukotrienes at the cost of the 2-series prostaglandins and 4-series leukotrienes [49,50,51]. Also, the CYP-derived metabolites of PUFA are shifted when increasing the *n*-3 intake, indicating that EPA and DHA are preferred over AA as substrates in this metabolic pathway [52,53]. However, in subjects with high basal seafood consumption, additional supplementation with fish oil did not alter the FA composition of phospholipids [54].

In addition, biochemical markers of inflammation have been shown to be affected by increased intake of *n*-3 FA. Plasma levels of the cytokines IL-6 and TNFα, along with the acute phase inflammatory protein *C*-reactive protein (CRP), have been shown to decrease with increased *n*-3 intake in both healthy people [55,56,57] and in patients with CVD [58,59,60].

As described in Section 2, dyslipidemia is recognized as a risk factor for development of atherosclerosis. Most clinical studies show that an increased intake of *n*-3, either as fatty fish or as supplements, decrease the plasma content of TAG [61,62,63,64]. Trends regarding cholesterol changes are not as conclusive. Several studies have shown a slight increase in LDL cholesterol levels following fish oil or *n*-3 supplementation [65,66]. It has been suggested that DHA, and not EPA, could be responsible for this phenomenon [67]. Other studies find no change in the LDL cholesterol content [61,63,64,68], yet others report a decreased LDL cholesterol level following increased intake of *n*-3 FA [69,70].

The effects of increased *n*-3 intake on blood pressure reduction are also inconclusive. Although some studies report an attenuation of blood pressure related to n-3 intake [71,72], others find no effect [73]. Systematic reviews on fish oil supplements and hypertension by Campbell *et al.* [74] and Miller *et al.* [75] show a modest improvement on blood pressure in hypertensive patients, but little or no effect on normotensive subjects.

#### Dietary Intake of Oxidized Lipids

Most of the existing literature on cardiovascular impact of intake of oxidized lipids is based on animal trials. Feeding mice with either oxidized *n*-3-rich PL or oxidised *n*-3-rich TAG resulted in increased plasma concentrations of 4-HHE and the inflammation markers IL-6 and MCP-1 compared to control groups receiving un-oxidised PL or TAG [76]. Other animal feeding trials with oxidized oils have shown reduced growth and increased production of inflammatory markers, and these effects were reduced with concomitant intake of antioxidants [77,78,79]. Yet other studies have reported a significant reduction of atherosclerotic plaques in animal models with simultaneous intake of antioxidants and marine oils [80,81].

Concerning the potential risks of human consumption of oxidized *n*-3, the available literature is scarce. Ottestad *et al.* [82] published a study showing no adverse effect of short-term intake of lightly oxidized oils in healthy volunteers. García-Hernandez *et al.* [83] came to the same conclusions for intake of lightly oxidized oils, while the conclusion for highly oxidized oils was that they have adverse effects on cholesterol levels. The duration of both these studies were, however, relatively short and the number of included subjects were low. Due to the suspicion of adverse biological effects of oxidized lipids, studies over a longer time-span and with more participants would be considered un-ethical and hence, such studies would be impossible to conduct.

## 4. Seafood or *n*-3 Supplements?

Fish and other seafood are the most important dietary sources of *n*-3 FA. Moreover, most marine species are low in *n*-6 FA, which make the *n*-6/*n*-3 ratio very low. As most seafood are subjected to some kind of household preparation prior to consumption, a range of studies focusing on retention of *n*-3 FA as a result of preparation have been performed. Most of them conclude that the absolute changes in *n*-3 content are low for most preparation techniques. However, when fat is added in the process, for instance in pan frying or deep frying, the total lipid content and the *n*-6/*n*-3 ratio is increased [84,85,86,87]. In addition to the *n*-3 FA, fish and seafood contain a wide range of other important nutrients, such as high-quality proteins, vitamin A, D, B_12_ and several minerals [88]. Seafood is also rich in the sulfur-containing free amino acid taurine, which has been shown to hold a range of cardioprotective properties [61,89]. Also other endogenous compounds, such as anti-oxidative peptides protecting the *n*-3 PUFA from peroxidation are embedded in the seafood [90]. Combinatory effects between *n*-3 FA and other beneficial compounds should therefore not be ruled out [91].

Crude fish oils are rich in *n*-3 FA, but they also contain other substances such as free fatty acids, phospholipids, pigments, sterols, metals and persistent organic pollutants (POP). Some of these are detrimental for health (such as POP) or for the quality of the oil. The presence of transition metals will for instance initiate lipid peroxidation. The removal of unwanted compounds by refining processes is thus necessary. Traditional refining involves several processes, including degumming, neutralization, bleaching and deodorization (steam distillation) and some of these are performed at high temperatures. During the refining process, also some desirable compounds, such as natural antioxidants, are partly removed or destroyed, reducing the overall cardioprotective effect of the fish oil. A gentler refining process, based on short-path distillation, has been developed [92] and this process has been shown to effectively remove unwanted compounds, but at the same time protect the desirables [93].

Fish oils will, however, still be prone to oxidation and different protective measures are therefore applied in order to inhibit, reduce or delay this process. Addition of antioxidants is one of these measures and this is done routinely in most, if not all, *n*-3 supplements. This certainly reduces and delays the peroxidation process, however, it will not be completely inhibited [94,95]. Encapsulation of the oils is another measure that has been shown to increase stability. Here, the fish oils are embedded in materials such as polydextrins and different proteins [96,97]. However, some encapsulation materials have been shown to mask odour from volatile oxidation products and this may reduce the ability to detect early stages of oxidation in these products [98]. Using high quality oils, with low lipid oxidation levels, as source material is therefore very important.

A wide range of *n*-3 supplements are on the market today. Most of these are sold as either traditional liquid fish oils, encapsulated fish oils or concentrated supplements. In liquid fish oils and some encapsulated *n*-3 supplements, the lipids are present as TAG and the content of EPA + DHA is normally around 30%. In the refining process of the oils, TAG may be trans-esterified with ethanol making FA ethyl esters (FAEE). The mixture of FAEE may be fractionated and up-concentrated, increasing the concentration of the targeted FA (EPA + DHA) up to around 90% [99]. This increase in *n*-3 PUFA in the supplements will in itself lead to higher risk of oxidation. In addition, in a recent study, FAEE oils have been shown to oxidize more rapidly than TAG oils [100].

## 5. Epidemiological Studies on Consumption of Fish and *n*-3 Supplements

Numerous studies have been performed in order to investigate beneficial effects following consumption of these FA, as fish oils, capsules or seafood [101,102,103,104,105,106,107,108]. Fish and other seafood are the most important dietary sources of *n*-3 FA and has traditionally been target for most of these studies (Table 1). Most such studies conclude that increased seafood consumption is beneficial and may contribute to a prevention of development of CVD, particularly in the reduction of fatal CVD events [102,106,108]. Another aspect of high seafood consumption is that it displaces other nutrient sources, with possible adverse effects on cardiac health, in the diet. For instance, Bernstein *et al.* [109] showed that one serving a day of fish instead of red meat was associated with 24% lower risk of CHD.

**Table 1:** Recent systematic reviews and meta-analyses evaluating the association between the consumption of fish and risk of cardiovascular disease.

Reference	Year	Main Conclusions
Zheng *et al.* [108]	2012	Included 315,812 participants; average follow up period 15.9 years. Low (1 serving/week) or moderate fish consumption (2–4 servings/week) had a prevented of CHD mortality significantly. Difficult to conclude for high fish consumption (>5 servings/week) due to the limited amount of studies of high fish consumption.
Djousse *et al.* [110]	2012	Included 176,441 participants; average follow up period 13.3 years. The authors concluded that there was a linear and inverse association between consumption of fish, as well as marine ω-3 fatty acids, and the risk of heart failure (HF).
Chowdhury *et al.* [111]	2012	Included 794,000 participants from 26 prospective cohort-studies and 12 randomized controlled trials; Higher (especially fatty) fish consumption (1 fish meal per week *vs.* 2 or more weekly meals) is moderately but significantly associated with a reduced risk of cerebrovascular disease. No association with marine *n*-3 fatty acids and cerebrovascular disease. Effect attributed to other components in fish?
Li *et al.* [112]	2013	Included 170,231 participants; average follow up period 9.7 years. Reported a dose-dependent inverse relationship between fish consumption and incidence of HF. Weekly fish consumption (once or more) could reduce HF incidence.
Xun *et al.* [113]	2012	Included 402,127 individuals with an average 12.8 years of follow-up. Observed a modest inverse association between fish intake and ischemic stroke.
Yinko *et al.* [114]	2014	Included 408,305 participants from 11 prospective cohort and 8 case-control studies and reported an inverse, dose-related association between fish consumption and the risk of acute coronary syndrome.

In recent years substantial efforts have been made to evaluate the accumulated effects on cardiovascular disease of fish consumption and of marine *n*-3 supplementation using systematical reviews and meta-analyses. A summary of these efforts is presented in Table 2.

**Table 2:** Recent systematic reviews and meta-analyses evaluating the association between intake of marine *n*-3 supplements and risk of cardiovascular disease.

Reference	Year	Main Conclusions
Musa-Veloso *et al.* [115]	2011	Included 214,426 subjects and concluded that a daily supplementation with 250 mg EPA and DHA (or more) significantly reduced the risk of sudden cardiac death, whereas the risks of total fatal coronary events and non-fatal myocardial infarction was not significantly reduced.
Delgado-Lista *et al.* [116]	2012	Included 46,737 subjects with high cardiovascular risk. The marine fatty acids EPA and DHA decreased the risk of cardiovascular events, cardiac death and coronary events, especially in patients with high cardiovascular risk, but no reduced risk of all-cause mortality.
Rizos *et al.* [117]	2013	Included 68,680 patients. The authors concluded that there was no association between EPA and DHA supplementation and lowered risk of all-cause mortality, cardiac death, myocardial infarction and stroke.
Casula *et al.* [118]	2013	Including 15,348 patients with a history of cardiovascular disease. Long-term supplementation of high dose *n*-3 PUFA may be beneficial for the onset of cardiac death, sudden death and myocardial infarction among patients with a history of CVD.
Zheng *et al.* [119]	2014	Included 24,788 patients with impaired glucose metabolism (IGM). EPA and DHA had no protective effect on cardiovascular mortality, major cardiovascular events or all-cause mortality in IGM patients.
Wen *et al.* [120]	2014	Included 32,656 patients with coronary heart disease (CHD). Supplement of *n*-3 PUFA in patients with CHD is associated with a reduction in death from cardiac causes, sudden cardiac death and death from all causes, whereas no association where found on major cardiovascular events.
Enns *et al.* [121]	2014	Included 396 patients with peripheral arterial disease. Not sufficient evidence to indicate a beneficial effect of *n*-3 PUFA supplementation on the incidence of cardiovascular events and related serous complications in adults with peripheral arterial disease.

Over the last few decades, *n*-3 FA has been generally considered effective means for lowering the risk of CHD and for secondary prevention of myocardial infarction (MI), effects that have been at least partially, ascribed to the previously discussed TAG-lowering effect. The DART randomized controlled trial (RCT) was the first RCT demonstrating that intervention with *n*-3 FA supplementation or increased intake of fish (advised to take fish oil capsules containing 500–900 mg *n*-3 FA/day or to eat 300 mg fatty fish per week), reduced the all-cause mortality in patients who had recovered from an earlier MI [122]. A few years later, the Italian GISSI-Prevenzione study consolidated these results in patients who recently had an MI. 11,324 patients where included and those receiving 885 mg EPA + DHA for up to 3.5 years had a 15% reduced combined risk for all-cause death and non-fatal cardiovascular events [123]. Also in the more recent, very large Japanese JELIS-study, a significant reduction was observed for the risk of coronary events in hypercholesterolemic patients receiving a daily dose of 1800 mg EPA in combination with 5 mg/day simvastatin compared to patients receiving statin only [107].

The apparent beneficial effect of *n*-3 PUFA supplementation on CVD was still evident a few years ago when the effects of marine *n*-3 supplementation was evaluated in patients with high cardiovascular risk [116], as EPA and DHA intake as food or supplement effectively prevented cardiovascular events, cardiac deaths and coronary events in patients with high cardiovascular risk [116]. Similar results were also indicated in the general population a few years ago, where those consuming more than 250 mg/day *n*-3 PUFA had a significant reduction in the risk of sudden cardiac death compared to subjects with a daily intake of less than 250 mg *n*-3 FA. On the contrary, Rizos *et al.* [117] presented a highly debated paper containing a cumulative meta-analysis of studies from 1995 to 2012 on *n*-3 supplementation and all-cause mortality. In this paper it was pointed out that the positive effect of *n*-3 supplementation has declined over the years, and that there no longer is a significant positive effect of *n*-3 supplementation on major cardiovascular outcomes. Their overall conclusion was that there are no longer grounds for recommending supplementary *n*-3 intake for protection against development of CVD. There are several possible explanations to why the positive effects of *n*-3 supplementation seem to decline and some of these issues will be discussed in the following.

The most frequently used experimental method in these studies, RCT, is traditionally recognized as the “gold standard” of epidemiological studies. In such studies participants are randomly divided into groups, usually one control group and one or more treatment groups. However, when conducting RCT certain assumptions have to be made, for instance that the basal diets of the participants in each group are similar. Over time these basal conditions may change and a meta-analysis comparing RCT over a long time-span may be affected by these changes. For instance, the seafood consumption has more than doubled throughout the world during the last decades, from an average of 9.9 kg·capita^−1^·year^−1^ in the 1960s to 17.0 kg in the 2000s and 18.9 kg in 2010. Preliminary estimates for 2012 are pointing towards further growth to 19.2 kg·capita^−1^·year^−1^ [124]. This fact alone could have reduced the potential benefits of supplementary *n*-3 [54,125].

Over the years the use of medical treatment for control of risk factors, such as statins for hypercholesterolemia and ACE-inhibitors for hypertension, has increased, and such medications may also diminish the effects of supplementary *n*-3. An interesting example of this was observed in the recent Italian randomized controlled clinical trial examining the effect of *n*-3 FA (EPA and DHA ethyl esters in a daily dose of 850 mg) on non-fatal MI, non-fatal stroke and death [104]. The observed incidents of these primary outcomes was much lower than anticipated, and after a year the study group had to change the outcome to hospitalization or death from cardiovascular causes. There may be several explanations for this observation, and this is obviously associated with the improved pharmacological interventions (such as the statin effect) in combination with a substantial improved acute treatment of acute coronary events ultimately resulting in a lowered death rate from cardiovascular causes.

Choosing the right control, or placebo, group is one of the most challenging factors of performing such studies. In several of the studies included in Rizos *et al.* [117], olive oil has been chosen as placebo [104]. Olive oil is rich in the monounsaturated FA oleic acid (C18:1, *n*-9), along with a range of polyphenolic compounds. Olive oil is therefore recognized as an effective anti-oxidant and may be protective against development of CVD in itself [126] and is hence, not the best choice as placebo when examining cardioprotective effects.

Most studies involving *n*-3 supplements are currently not reporting the degree of oxidation of the supplements used in the trials [127]. As discussed in Section 4, the quality and stability of *n*-3 supplements may be important factors when evaluating their cardioprotective effects. Recently a range of studies on the oxidative status on over-the-counter *n*-3 supplements have been published showing that the contents of *n*-3 are lower than declared and that the limits for oxidation products are exceeded in a large number of products [128,129,130,131]. Inferior quality of the supplements may therefore be another explanatory factor to the lack of evidence for positive effects of *n*-3 supplementation. Several recent meta-analyses and systematic reviews have presented results using different patient populations without finding any significant protective effects from EPA and DHA on major cardiovascular events, MI, cardiac death or all-cause mortality [119,121]. However, in patients with a history of CVD or CHD, *n*-3 PUFA supplementation may still lower the risk for MI and sudden cardiac death [118,120].

## 6. Dietary Recommendations

In a study by Mozaffarian and Rimm [125], the correlation between *n*-3 intake and protective effect was reviewed. Their conclusion was that a modest intake of fish, *i.e*., 1–2 servings per week corresponding to approximately 250 mg EPA + DHA per day, reduced the risk of coronary death by 36% and risk of total death by 17%. A higher seafood intake did not reduce the risk further.

Another focus regarding protective effects of *n*-3 FA is the relationship or ratio between *n*-6 and *n*-3 FA in the diet. In historic times, the ratio between these two FA was around 1:1, while in present times this ratio has shifted markedly in favor of *n*-6. Current ratios are 4:1 in Japan, 15–17:1 in the Western societies (Europe and USA) and 38–50:1 in urban areas of India [132]. The imbalance between these FA as seen worldwide today may promote the development of several lifestyle-related diseases such as CVD [132,133,134,135]. Increasing seafood intake is considered to be a good strategy in order to achieve an improved ratio between these FA.

In 2010 and 2014, two large analyses of the risks and benefits of consuming seafood were performed, one by FAO/WHO [136] and one by the Norwegian Scientific Committee for Food Safety [91]. The conclusion in both of these studies was that adults consuming less than one serving of seafood per week may miss the beneficial effects of seafood on CVD.

Based on the conclusions from the epidemiological studies on seafood consumption and the risk and benefit analyses, several organizations and authorities have published dietary recommendations regarding intake of marine *n*-3 FA and/or fish (Table 3).

**Table 3:** Overview of dietary recommendations regarding fish or marine *n*-3 fatty acids.

Authority/Organization	Country/Region	Year	Recommendation	Reference
The American Heart Association	USA	2015	A variety of (preferably fatty) fish at least twice a week	[137]
The Norwegian Directorate of Health/VKM	Norway	2014	Fish as dinner at least 2–3 times per week	[91]
Food and Agricultural Organization of the United Nations (FAO)/World Health Organization (WHO)	World	2011	At least 1–2 100 g servings of fatty fish per week	[136]
European Food Safety Association (EFSA)	Europe	2010	250 mg EPA + DHA daily	[138]
Scientific Advisory Committee for Nutrition (SACN)	UK	2004	450 mg EPA + DHA daily	[139]
International Society for the Study of Fatty Acids and Lipids (ISSFAL)	UK/Europe	2004	500 mg EPA + DHA daily	[140]

Table 3 shows the recommendations given to general, healthy populations. Several authorities/organizations specify that people with documented health issues, such as CVD, may need more *n*-3 than the general population. For instance, AHA recommends two weekly seafood meals for people without documented CHD, 1 g EPA + DHA for people with documented CHD and 2–4 g EPA + DHA for people in need for lowering TAG [137].

The more recent official advice focus on intake of fish and seafood as sources of *n*-3 FA. This is partly because uptake and incorporation of *n*-3 from seafood has been shown to be superior to that from *n*-3 supplements [141,142,143]. Another reason for recommending seafood intake over *n*-3 supplements, as stated in Section 4, is that seafood, in addition to its *n*-3 content, is also a rich source of other nutrients that may provide synergy effects towards health benefits.

## 7. Conclusions

Fish and seafood are the optimal sources of *n*-3 FA and there is a broad consensus that the general consumption of seafood should be approximately 2–3 servings per week in order to obtain the maximal protective effect towards development of CVD. There are conflicting findings between no effects *vs.* beneficial effects in recent RCT and meta-analyses for *n*-3 supplements. The scientific evidence for benefits from *n*-3 supplementation has diminished over time, probably due to a general increase in seafood consumption and better pharmacological intervention and acute treatment of patients with CVD.
